# Resolution of Ascending Aorta and Pulmonary Vein Thrombi With Apixaban Therapy: A Case Study

**DOI:** 10.7759/cureus.92269

**Published:** 2025-09-14

**Authors:** Hidekazu Takeuchi

**Affiliations:** 1 Internal Medicine (Cardiology), Takeuchi Naika Clinic, Ogachi-Gun, JPN

**Keywords:** acute ischemic stroke (ais), acute myocardial infarction (ami), ascending aorta (aao) thrombi, pulmonary vein thrombi, right upper pulmonary vein thrombi, transesophageal echocardiography

## Abstract

Objective: This study aimed to investigate ascending aorta (AAo) thrombi and the effects of apixaban using transesophageal echocardiography (TEE) and cardiac computed tomography (CT).

Introduction: Studies of thrombi retrieved from acute ischemic stroke (AIS) patients revealed that those thrombi contained collagen and calcifications, indicating that the thrombi were chronic or old. These patients had chronic thrombi before AIS occurred, and some of the thromboemboli could separate from chronic thrombi and cause AIS. Left atrial appendage (LAA) thrombi in atrial fibrillation (AF) patients are candidates for such chronic thrombi; however, patients without LAA thrombi may have an AIS. Where do those thrombi come from? In prior reports, we described several cases of pulmonary vein thrombi (PVTs), which can cause AIS and acute myocardial infarction (AMI) by separating large clots. We found other candidates in the AAo. We found that a reduced dose of apixaban partially resolved AAo thrombi.

Patient presentation: A 73-year-old male with hypertension was examined using cardiac CT and TEE to detect AAo thrombi and PVTs. The patient was treated with apixaban.

Results: TEE revealed white thrombi with surrounding dark thrombi on the right side in the AAo, and the white thrombi seemed to connect to white right upper pulmonary vein (RUPV) thrombi through line-like white thrombi. A reduced dose of apixaban (2.5 mg; twice daily) was used because the dose reduction weight criteria were met and resolved most AAo thrombi; however, the decreased dose of apixaban (2.5 mg; once a day) was not able to prevent AAo thrombi from regrowing.

Discussion: TEE demonstrated that RUPV thrombi, seemingly contacting line-like white thrombi, and thrombi in the AAo were affected by apixaban. Line-like white thrombi looked to approach the wall of the AAo, and approaching areas looked like a mass; the wall of the AAo could not be clearly identified. How the wall was affected is unknown and could be associated with leukocytes such as monocytes, macrophages, and myofibroblasts present in white thrombi with shadows around the ostia of the RUPV.

Conclusion: White RUPV thrombi approached AAo thrombi through white line-like thrombi. These thrombi were partially resolved by treatment with a reduced dose of apixaban; however, the positive effect diminished with decreasing dose.

## Introduction

Research on thrombi retrieved from acute ischemic stroke (AIS) patients has shown that those thrombi have collagen and calcifications [1-4], indicating that they are chronic or old. Doctors who treat AIS typically use the term cardiogenic embolization; however, where the thrombi originate is generally unclear. Left atrial appendage (LAA) thrombi are chronic thrombi and are thought to be the source of cardiogenic thrombi in atrial fibrillation (AF) patients. Many patients without AF are affected by AIS, and where those thrombi originate from is unclear.

Previously, using cardiac computed tomography (CT) and transesophageal echocardiography (TEE), we described several cases of pulmonary vein thrombi (PVTs) and reported that PVTs are common in age-related diseases and that warfarin or direct oral anticoagulants (DOACs) partially resolved PVTs [5-12]. Compared with warfarin, DOACs are known to have similar effects and fewer side effects. In addition, we reported that PVTs and left atrium (LA) thrombi extending from PVTs have calcifications and are heterogeneous, indicating that those thrombi could cause AIS and acute myocardial infarction (AMI) [12]. Recently, we identified new types of thrombi in the ascending aorta (AAo) using TEE; these thrombi may be alternative candidates for chronic thrombi that cause AIS and AMI. To determine the relationships between right upper pulmonary vein (RUPV) thrombi and AAo thrombi, TEE and cardiac CT were used.

## Case presentation

A 73-year-old male with hypertension was examined using cardiac CT and TEE to assess the presence of chronic thrombi, such as LAA thrombi and PVTs. He had no symptoms of paralysis, chest pain, or exertional dyspnea in daily life. His blood pressure was 136/85, his heart rate was 65 bpm, and his physical examination was unremarkable. The patient was 167 cm tall and weighed 59 kg; thus, his BMI was 21.2 kg/m^2^. His brain natriuretic protein (BNP) concentration was 21.2 pg/mL (normal; <18.4 pg/mL); however, his serum creatinine concentration was 0.62 mg/mL (normal; 0.65-1.07 mg/mL), and his urine albumin creatinine ratio (ACR) was 4.1 mg/gCr (normal; <30 mg/gCr). He had not been previously treated with warfarin or DOACs.

Electrocardiography (ECG) demonstrated counterclockwise rotation, normal sinus rhythm, a normal axis, no ST-T changes, and no notching. AF and structural heart disease were ruled out. The serum D-dimer concentration was 0.6 μg/ml (normal; < 1.0 μg/ml), the activity of protein S was 90% (normal; 74-132%), and the activity of protein C was 104% (normal; 64-135%). The homocystein concentration was 7.4 nmol/mL (normal; 5-15 nmol/mL).

**Figure 1:**
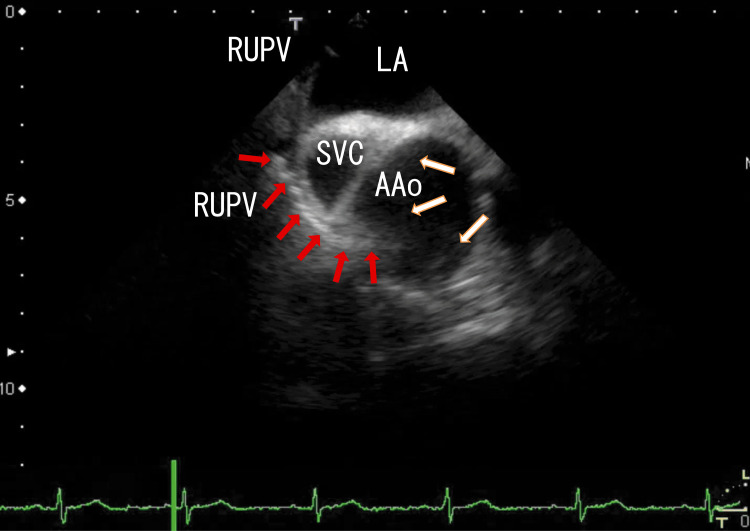
Transesophageal echocardiography (TEE) images showing ascending aorta (AAo) thrombi TEE revealed white right upper pulmonary vein (RUPV) thrombi, white line-like thrombi located under the superior vena cava (SVC), and AAo thrombi (red arrows). The white line-like thrombi reached near the wall of AAo; those areas were dim, and the structure of the LA wall was unclear. AAo thrombi appeared as white lines and dim, dark white masses around the white lines (white arrows) on the right wall of the AAo. The approximate thrombus size was 4 cm × 3 cm. AAo: ascending aorta, LA: left atrium, RLPV: right lower pulmonary vein, RUPV: right upper pulmonary vein, SVC; superior vena cava

TEE revealed white thrombi around the ostia of the RUPV, which seemed to be connected to line-like white thrombi situated on the side of the superior vena cava (SVC) and reached white thrombi in the AAo (Fig. 1). There were rather vague, large, whitish thrombi around the central white thrombi in the AAo (Fig. 1) (start). Video images revealed that the AAo thrombi moved with heartbeats; however, the RUPV thrombi and line-like thrombi did not move with heartbeats (Video 1) (start). Cardiac CT did not reveal AAo thrombi, which are similar to LA thrombi that extend from PVTs. Previously, we reported several cases of LA thrombi extending from PVTs. Cardiac CT was able to identify PVTs in the pulmonary vein; however, those LA thrombi could not be identified by cardiac CT, which might have been caused by special resolution limitations or the timing of contrast phases. This finding has implications for diagnostic pathway recommendations.

**Video 1:** TEE images showing RUPV thrombi directly connected to the thrombi in the AAo TEE images revealed white RUPV thrombi that extended as line-like white thrombi and reached the wall of AAo, where white parts of AAo thrombi were present. The white line-like thrombi appeared to be continuous from RUPV thrombi to AAo thrombi. The white line appeared to penetrate the right wall of the AAo in Figure 1. The AAo thrombi were very mobile in sync with the heartbeats; however, the RUPV thrombi and extended white line-like thrombi did not move with the heartbeats. Therefore, the white line thrombi did not penetrate the AAo wall. The structure of the right wall of the AAo was not clear. RUPV thrombi had white shadows, indicating that they involved calcifications. The angle of this video is the same as that in Figure 1.

**Figure 2:**
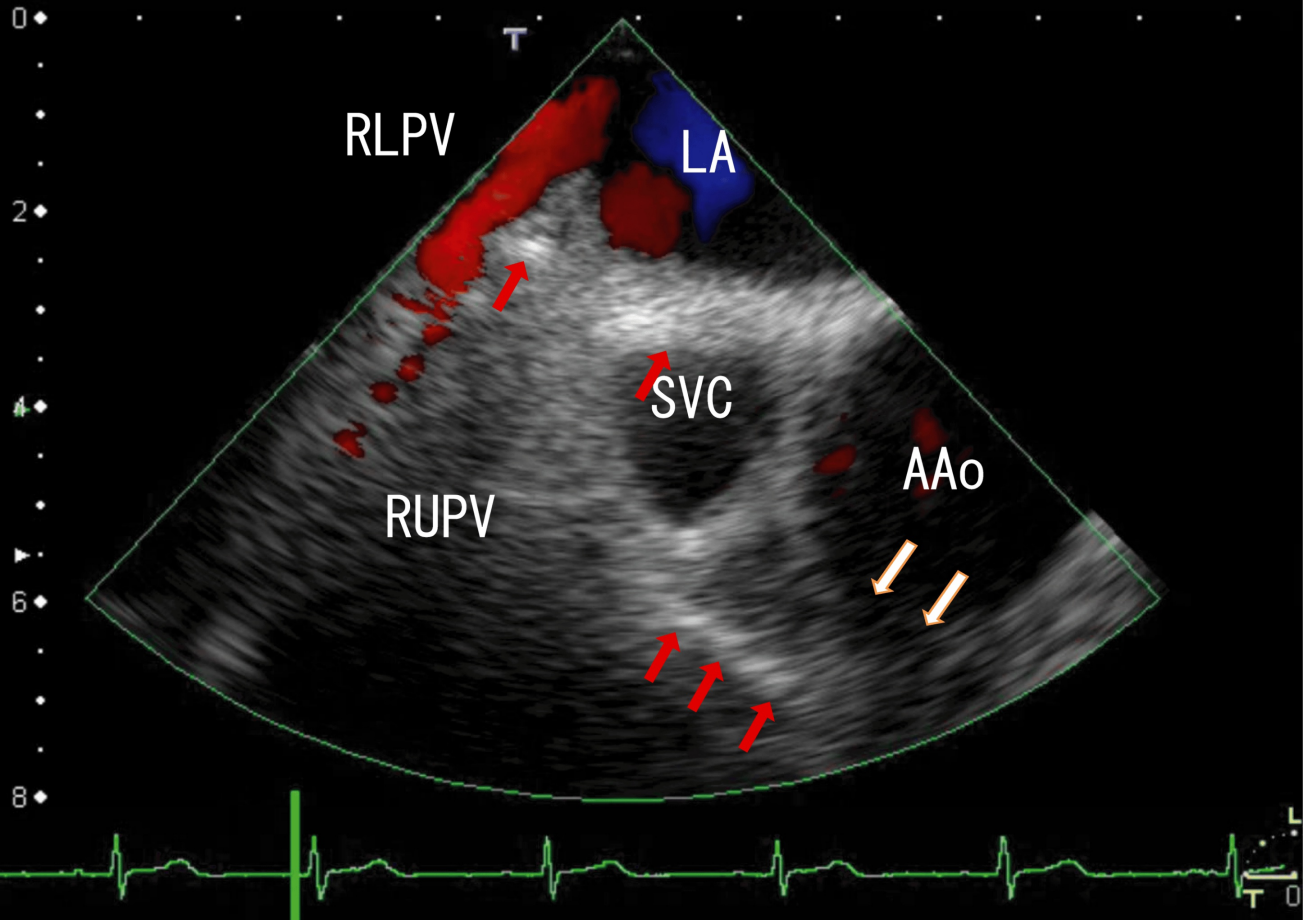
After six months of reduced-dose apixaban treatment, the TEE images revealed that most AAo thrombi had resolved. After six months of reduced-dose apixaban treatment, the TEE images revealed that most AAo thrombi resolved except those in the lower parts of the AAo. Additionally, the right side of the line-like white thrombi disappeared. The approximate thrombus size was 1 cm × 0.5 cm. AAo: ascending aorta, LA: left atrium, RLPV: right lower pulmonary vein, RUPV: right upper pulmonary vein, SVC; superior vena cava

We treated the patient with a reduced dosage of apixaban (2.5 mg; twice daily) for the next six months because the patient weighed 59 kg and the dose reduction weight criteria were met. Most AAo thrombi resolved, and the approaching line-like thrombi appeared unclear (Fig. 2, Video 2) (six months passed from the start). The serum D-dimer concentration was 0.5 μg/ml, and his serum creatinine concentration was 0.79 mg/mL.

**Video 2:** After six months of apixaban treatment, the TEE images revealed that most AAo thrombi had resolved. After six months of treatment with a reduced dose of apixaban, the TEE images revealed that most AAo thrombi resolved, except for the lower parts of the AAo thrombi. The AAo thrombi were mostly resolved; we could not identify the wall of the AAo at the AAo attachment areas. A mass of AAo thrombi and walls approached white line-like areas that did not move with heartbeats. We could not find the missing right side of the connecting thrombi. The blood flow from the right middle pulmonary vein (RMPV) is shown as a red area. The angle of this video is the same as that in Figure 2.

**Figure 3:**
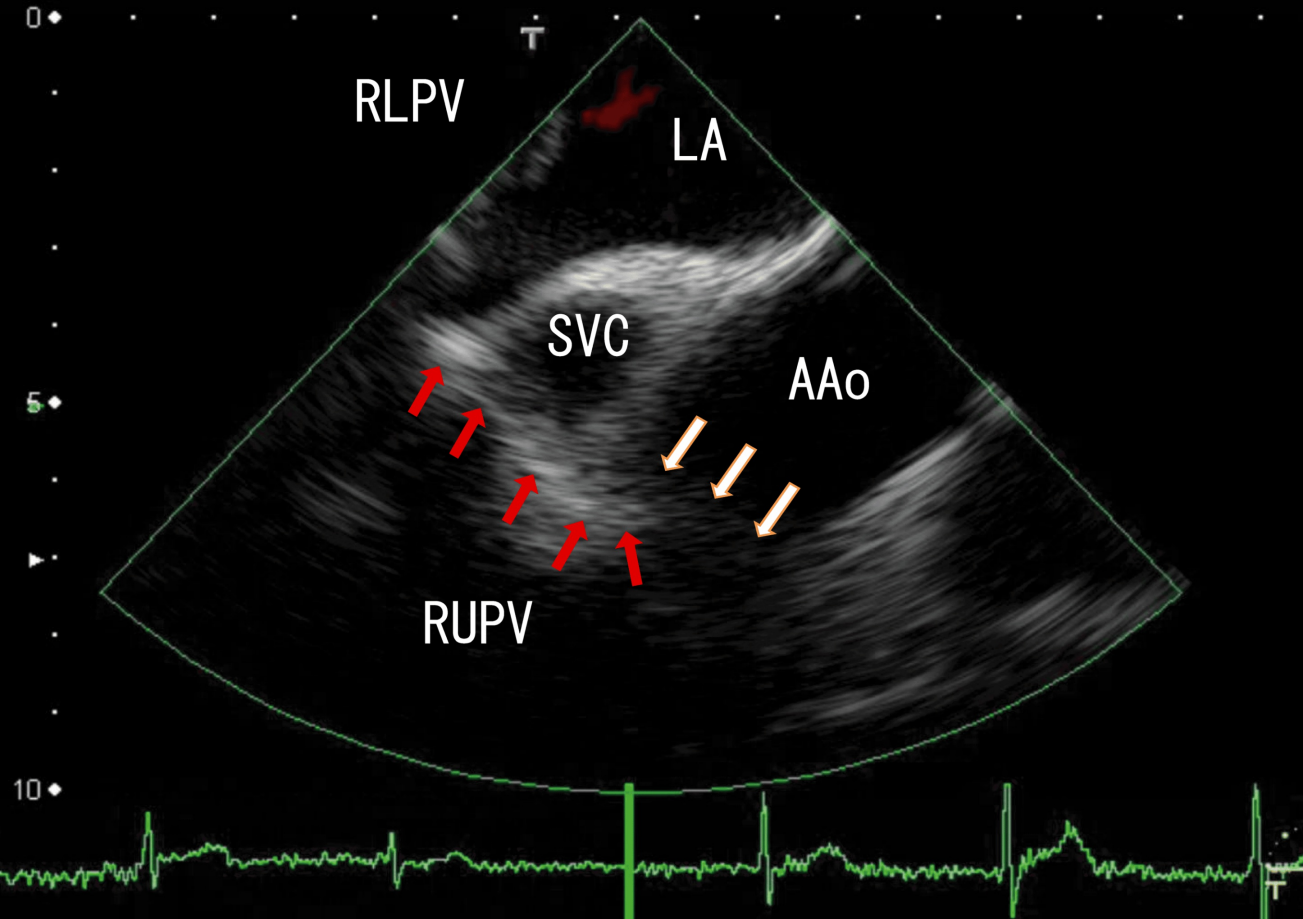
After six months of decreased-dose apixaban treatment, TEE revealed growing AAo thrombi. After the next six months of decreased-dose apixaban treatment, TEE revealed the generation of small AAo thrombi. Additionally, the missing part of the right side of the connecting thrombi reappeared as thin line-like thrombi. The approximate thrombus size was 2 cm × 1 cm. AAo: ascending aorta, LA: left atrium, RLPV: right lower pulmonary vein, RUPV: right upper pulmonary vein, SVC; superior vena cava

For the next six months, we treated the patient with decreased dosages of apixaban (2.5 mg; once a day) to prevent side effects. Small, regrown AAo thrombi subsequently appeared, and thin connecting thrombi between RUPV thrombi and AAo thrombi appeared (Fig. 3, Video 3) (12 months passed from the start). If we used a half dose of apixaban (1.25 mg; twice daily) instead of 2.5 mg once a day, the results might be different. The serum D-dimer concentration was 0.5 μg/ml, and his serum creatinine concentration was 0.70 mg/mL.

**Video 3:** After six months of decreased-dose apixaban treatment, TEE revealed growing AAo thrombi. After six months of decreased-dose apixaban treatment, TEE revealed the generation of small AAo thrombi, which were white in the center. The AAo thrombi and the wall of the AAo made a mass, and the AAo thrombi appeared to connect to the RUPV thrombi through white line-like areas. Additionally, the missing part of the right side of the connecting thrombi reappeared as thin line-like thrombi. The angle of this video is the same as that in Figure 3.

**Figure 4:**
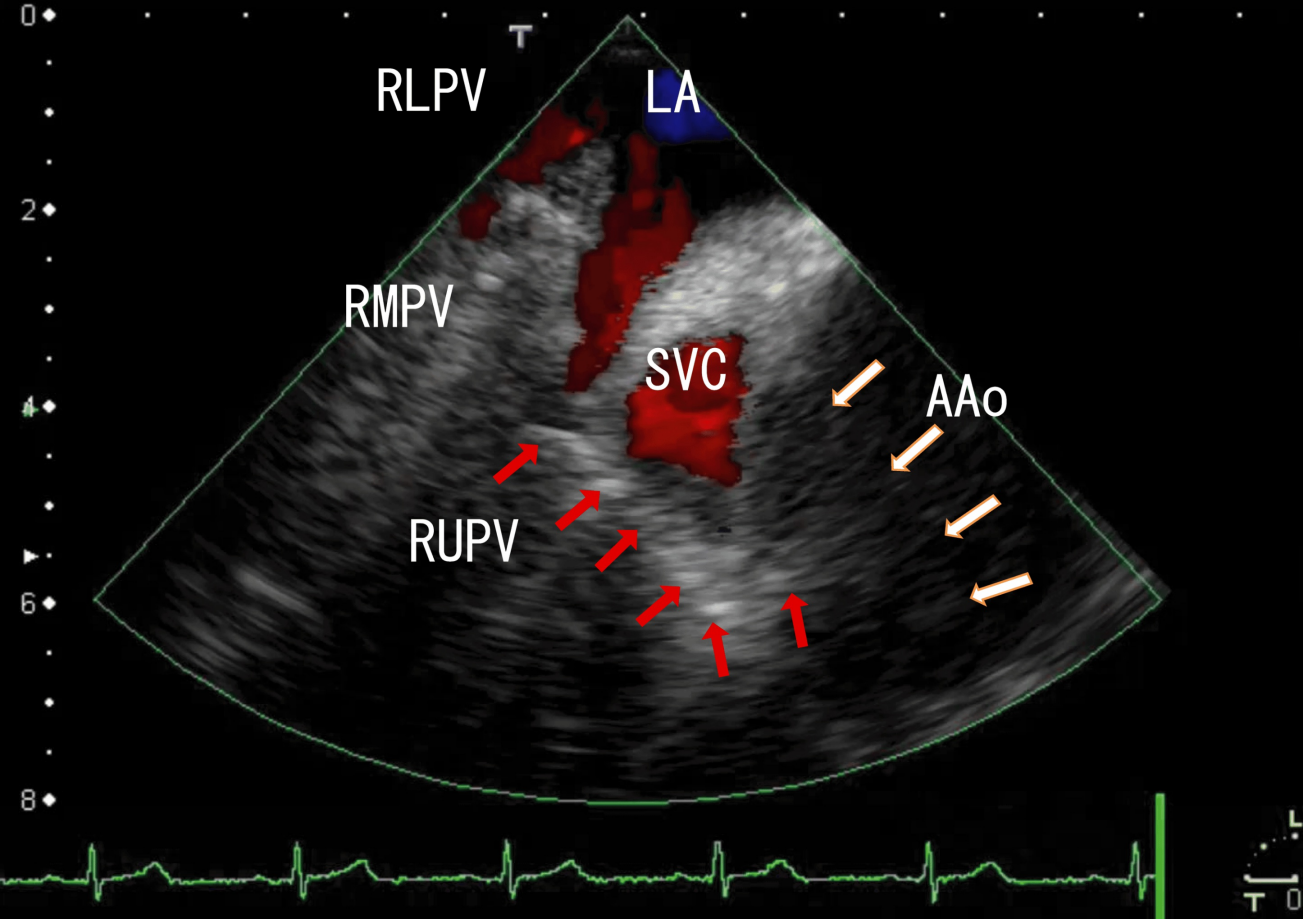
After 12 months of treatment with a decreased dose of apixaban, the TEE images revealed large AAo thrombi. After 12 months of decreased-dose apixaban treatment, TEE revealed the generation of large AAo thrombi. Additionally, the missing part of the right side of the connecting thrombi reappeared as line-like thrombi, which were similar to those in the first stage. The approximate thrombus size was 3 cm × 2 cm. AAo: ascending aorta, LA: left atrium, RLPV: right lower pulmonary vein, RMPV: right middle pulmonary vein, RUPV: right upper pulmonary vein, SVC; superior vena cava

For the following year, we continued treating the patient with decreased apixaban, and the TEE images directly demonstrated that the RUPV thrombi connected to the AAo thrombi through line-like white thrombi, which were similar to the images before treatment (Fig. 4, Video 4) (two years had passed since the start). The serum D-dimer concentration was 0.5 μg/ml, and his serum creatinine concentration was 0.78 mg/mL.

**Video 4:** After 12 months of decreased-dose apixaban treatment, the TEE images revealed large AAo thrombi. After 12 months of decreased-dose apixaban treatment, the TEE images revealed large AAo thrombi. The AAo thrombi are composed of thin white parts and large dark areas. The white parts looked like having two parts: one was moving with heartbeats, and the other was not moving with heartbeats. Additionally, the missing part of the right side of the connecting thrombi reappeared as line-like thrombi, which were similar to those before treatment. Several white thrombi located in the RMPV presented shadows, indicating that the thrombi were calcified. The angle of this video is the same as that in Figure 4.

These findings suggest AAo thrombus formation, which starts with AAo thrombi in Video 2, then in Video 3, and lastly, in Video 4. In Video 1, there were some moving thrombi on the left side of the AAo, which might have been thrombi extending from the right-side AAo thrombi.

No embolic events, such as AIS or AMI, were observed during follow-up, indicating that these treatment strategies have potential clinical safety.

The time course of the treatments is summarized in Table 1.

**Table 1:** Time course of treatment This table shows the time course of apixaban treatment and the changes in thrombus size and D-dimer levels.

Duration	Duration (total)	Treatment	Thrombus size	D-dimer
0 (start)	0 (start)	-	4 cm × 3 cm	0.6 μg/ml
6 months	6 months	apixaban (2.5 mg × 2)	1 cm × 0.5 cm	0.5 μg/ml
6 months	12 months	apixaban (2.5 mg × 1)	2 cm × 1 cm	0.5 μg/ml
one year	two years	apixaban (2.5 mg × 1)	3 cm × 2 cm	0.5 μg/ml

## Discussion

The RUPV thrombi approached the AAo thrombi via line-like white thrombi, which were previously unreported. Research on retrieved thrombi revealed that those thrombi include fibrin and collagen. After six months of treatment with a reduced dose of apixaban, some parts of white line-like thrombi disappeared; however, after the apixaban dose decreased to half, the white thrombi gradually increased, indicating that the white line-like areas could have been thrombi. The line-like white thrombi might be composed mainly of collagen, which could be affected by apixaban, indicating that those line-like white thrombi could be resolved, even when they existed outside vessels.

AAo thrombi can cause AIS and AMI as chronic thrombi, which can be inhibited by apixaban. Moreover, line-like white thrombi extending to RUPV thrombi seemed to affect the wall of the AAo, indicating the possibility that PVTs could affect the tissue around the heart, which could be repaired by apixaban. Line-like white thrombi could represent organized fibrin bridges, organized collagen bridges, or endothelial projections. Two types of thrombi are well known. One is thrombi made in the vessels and in the LA, and the other is thrombi made on the skin when we are injured. Here, we report the third type of thrombi, which were made between RUPV thrombi and AAo thrombi. Furthermore, the third type of thrombi, the line-like white thrombi, might have special characteristics in that they could interact with the wall of AAo. These comments are described here for the first time; however, more studies are needed to clarify these relationships.

Recently, two types of calcifications have been reported in retrieved thrombi; the large type has many CD45+/CD68+/α-SMA+ cells [13], which are known to become myofibroblasts [14,15]. Surrounding the entrance of the RUPV and right middle pulmonary vein (RMPV) are many white areas with strong white shadows (Video 1, Video 2, Video 4), indicating that the white areas with shadows may include large type calcified thrombi with many CD45+/CD68+/α-SMA+ cells, which are thought to be myofibroblasts. These CD45+/CD68+/α-SMA+ cells could become smooth muscle cells [16] and might interfere with the smooth muscle of the AAo wall; therefore, white thrombi might attach to the walls of the AAo. PVTs might directly affect surrounding tissues. To clarify these mechanisms, more studies are needed.

PVTs might cause AIS and AMI through the release of large clots. Therefore, small clots, including neutrophil extracellular traps (NETs), are separated and can cause some chronic diseases. NETs are reportedly associated with many diseases, such as cancer [17], cancer metastasis, central nervous system disease [18], atherosclerosis [19], and heart failure [20]. In 2015, we reported the use of PVTs in a patient with malignant lymphoma [6]. The patient was treated twice and experienced two recurrences. At present, we note that the rod-like large LA thrombi extending from the right lower pulmonary vein (RLPV) are very unique to this malignant lymphoma patient. LA rod-like thrombi extending from RLPV thrombi vibrated with heartbeats in the LA, indicating that the thrombi were free-end. Vibrating LA thrombi are not common and might produce more NETs. We reported that DOACs partially resolved rod-like LA thrombi extending from RLPV thrombi [7]. Therefore, we believe that before cancer treatment, treatment with DOACs might be beneficial. To clarify the association of NETs, it could be beneficial to identify any supporting biomarkers (e.g., MPO-DNA complexes) in future studies. More studies are needed to clarify these relationships.

We reported that RLPV thrombi were located in the LA and reached the anterior wall of the LA. We detected the left atrial diverticulum (LAD) around the attachment region [11] and an end-QRS notch and an early repolarization (ER) of their ECG [8], indicating that PVTs could induce changes in their attached tissues, including the walls of the LA and the AAo. Aortic valve calcification and stenosis might be associated with PVTs, because PVTs often coexist with AAo thrombi [9,10], and AAo thrombi expand to the opposite wall of the AAo in the present case. In the present case report, AAo thrombi changed the appearance of the wall of the LA by attaching to it, and RUPV thrombi extended the line-like thrombi to near the attachment area. In any case, AAo thrombi and PVTs may change the LA wall structure, which is unknown and should be studied further. Importantly, AAo thrombi can cause AIS and AMI as chronic thrombi, which can be inhibited by resolution using a reduced dose of apixaban; however, this was only one case. More studies are needed to verify these relationships.

Limitations

This article describes only one case; therefore, we cannot assert. Retrieved thrombi were not necessarily parts of PVTs and LA thrombi extending from PVTs, which are not directly proven. The images in this article were obtained by TEE; if possible, it would be better to use other modalities. These images of thrombi may be biased; however, most of the images seemed to be free from bias as a whole. The structural changes in the AAo wall are difficult to understand; myofibroblasts are just one candidate for associated factors, and other factors might actually play important roles. More studies are needed to confirm the information in the present case report and associated affairs.

## Conclusions

The white RUPV thrombi seemed to connect to the white AAo thrombi through the line-like white thrombi, and the LA wall near the AAo thrombi could not be identified. These thrombi were resolved by the reduced dose of apixaban; however, the decreased dose of apixaban was not able to prevent those thrombi from regrowing, and no embolic events were observed during follow-up. Cardiac CT did not reveal AAo thrombi, which are similar to LA thrombi that extend from PVTs.The white RUPV thrombi seemed to connect to the white AAo thrombi through the line-like white thrombi, and the LA wall near the AAo thrombi could not be identified. These thrombi were resolved by the reduced dose of apixaban; however, the decreased dose of apixaban was not able to prevent those thrombi from regrowing, and no embolic events were observed during follow-up. Cardiac CT did not reveal AAo thrombi, which are similar to LA thrombi that extend from PVTs.
